# Factors affecting anophthalmic socket reconstruction outcomes using autologous oral mucosal graft

**DOI:** 10.1186/s12886-024-03301-3

**Published:** 2024-04-04

**Authors:** Orapan Aryasit, Yanin Panyavisitkul, Parichat Damthongsuk, Penny Singha, Narisa Rattanalert

**Affiliations:** Department of Ophthalmology, Faculty of Medicine, Prince of Songkla University, 15, Kanjanavanich Road, Kohong, Hat Yai, Songkhla, 90110 Thailand

**Keywords:** Anophthalmic socket, Socket reconstruction, Oral mucosal graft, Prosthesis fitting

## Abstract

**Background:**

Limited studies have reported surgical outcomes that are defined by strict criteria following grade 2 or 3 socket reconstruction using an oral mucosal graft (OMG). We aimed to determine factors influencing surgical outcomes of anophthalmic socket reconstruction using OMG in patients with grade 2 or 3 socket contractures.

**Methods:**

Thirty-seven patients who underwent socket reconstruction with autologous OMG between January 2007 and December 2017 were retrospectively analyzed. The successful outcome was defined as an eye prosthesis wearing without experiencing displacement and the absence of any re-operations or additional surgeries following socket reconstruction. Factors affecting surgical outcomes were identified using multivariate analysis.

**Results:**

A total of 15 male and 22 female patients (mean age: 40.2 ± 17.2 years) were included. The median duration of socket contracture was 21.5 years. Grade 2 and 3 socket contractures, based on Tawfik’s classification, were reported in 20 and 17 patients, respectively. Twenty-eight and eight patients underwent socket reconstruction using OMG alone and OMG combined with a hard palate graft, respectively. The success rates of grades 2 and 3 socket contracture reconstruction were 80.0% and 52.9%, respectively. Multivariate analysis demonstrated that only grade 3 contractures were predictive of worse outcomes. At the final visit (mean follow-up: 6.3 years), 34 patients (91.9%) could wear their eye prostheses.

**Conclusions:**

Socket reconstruction using autologous OMG can provide acceptable results in grade 2 and 3 contractures; however, satisfactory results were more significantly reported in grade 2 than in grade 3 contractures.

## Background

Socket contracture is a major problem in patients with anophthalmia and is defined as the shrinkage and shortening of any part of the orbital tissue. It causes a decrease in the depth of the fornices and orbital volume, ultimately leading to the inability to retain an eye prosthesis [1]. There are various classifications of socket contractures according to severity. In 2009, Tawfik et al. proposed a novel classification system for management algorithms, categorizing socket contracture into four groups [2]. This classification system suggests a mucous membrane graft for socket reconstruction in advanced cases of grade 2 and 3 socket contractures [2, 3]. 

A mucous membrane graft is a substitute graft in which the need for healthy conjunctival epithelial cells to differentiate and multiply over the graft is obviated [2, 4]. The lower lip, upper lip, buccal mucosa, and hard palate mucosa can serve as donor sites for the oral mucosal graft (OMG). The hard palate mucosa is more rigid than the lip and buccal mucosa as it contains a perichondrium [5, 6]. It is known to provide superior results, particularly with the least contracted OMG and reduced shrinkage; however, it does have a rougher surface [5]. 

To our knowledge, limited studies have reported the surgical outcomes, which were defined by strict criteria, following grade 2 or 3 socket reconstruction using OMG. Therefore, we aimed to identify the success rate of surgical outcomes over the past 10 years and determine the associated prognostic factors.

## Methods

This retrospective study was performed at Songklanagarind Hospital, Prince of Songkla University, and was conducted in accordance with the criteria set by the Declaration of Helsinki. The study protocol was approved by the Human Research Ethics Committee of the Faculty of Medicine, Prince of Songkla University (REC. 61-028-2-4) and the need for informed consent was waived owing to the retrospective nature of the study.

The classification of socket contracture was defined as follows: grade 1 represents minimal contracture; grade 2 indicates mild contracture of the lower and/or upper fornix; grade 3 involves more extensive contracture than grade 2, rendering the patient unable to wear a prosthesis; and grade 4 signifies severe phimosis of both the upper and lower fornix, recurrent cases, or an irradiated socket [2]. We included only patients classified with grade 2 or 3 socket contractures who underwent socket reconstruction using either buccal or hard palate mucosal grafts between January 2007 and December 2017. Patients were excluded if they were followed up for less than 6 months. Electronic medical records, including demographic data, type and cause of eye removal surgery, onset of contracture, degree of socket contracture, additional surgery, the status of eye prosthesis fitting, and grading of surgical outcome, were reviewed.

**Fig. 1:**
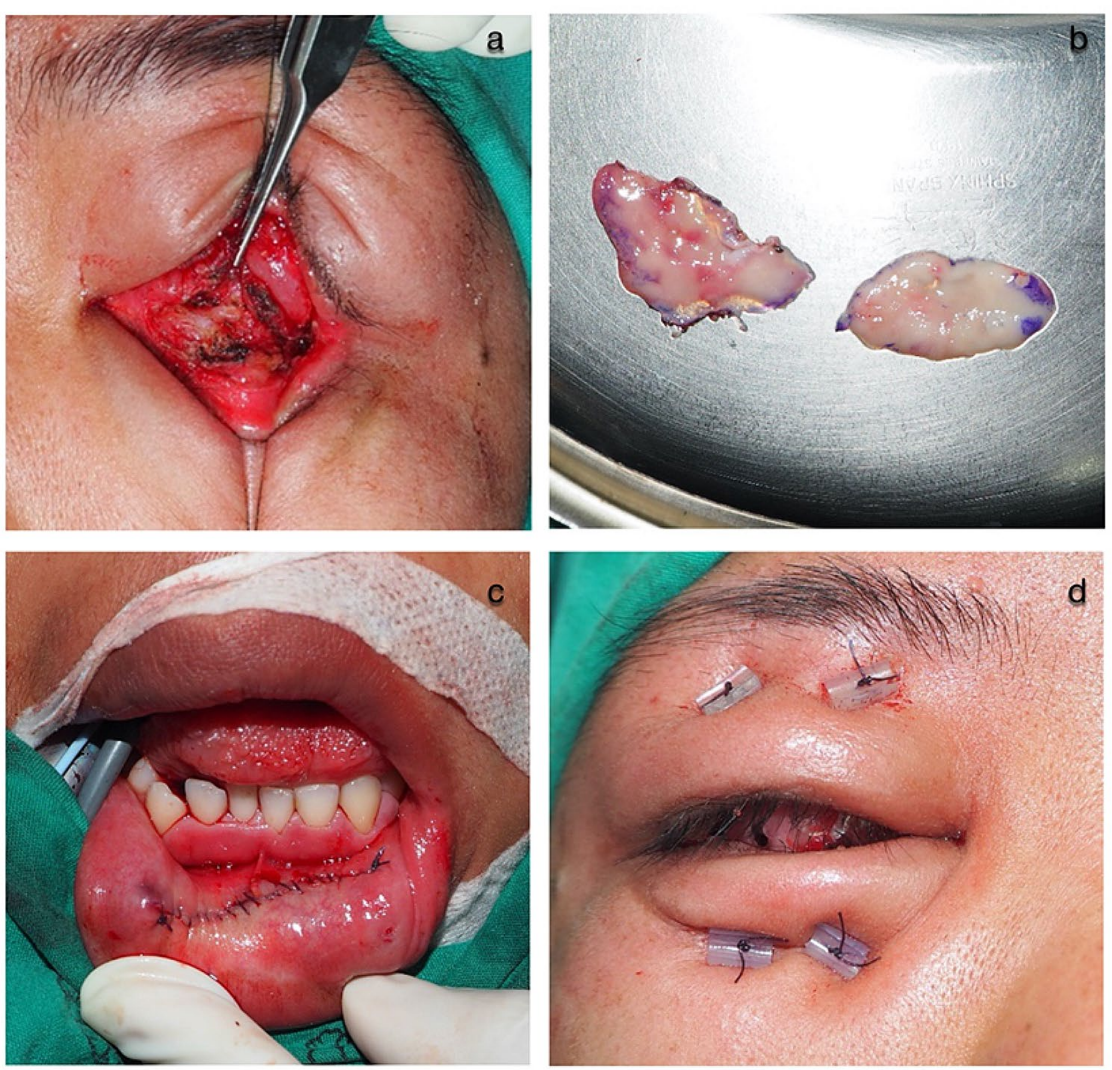
Surgical technique of socket reconstruction using autologous oral mucosal graft. **a** The inferior and superior conjunctiva was incised horizontally to create a cavity. **b** Harvested oral mucosal grafts from the inner aspect of the lower lip and the inner aspect of the cheek. **c** The donor site was closed. **d** Fornix fixation sutures were used to deepen the upper and lower fornix

All surgeries were performed under general anesthesia. Contracted conjunctival fornices were exposed using 5 − 0 silk traction sutures. The inferior (with or without superior) conjunctiva was incised horizontally and dissected to the orbital rim to create a cavity for the eye prosthesis. Full-thickness OMG was harvested from the inner aspect of the lower lip alone or along with the inner aspect of the cheek by adding 20% to the measurement of raw surface defects. The donor site was closed using continuous lock 4 − 0 absorbable braided polyglactin sutures. The submucosal tissue of the harvested OMG was removed using Wescott scissors. The OMG was placed on the raw surface defect at the fornix, and sutures were interrupted and continuously applied to the surrounding conjunctiva using 6 − 0 absorbable braided polyglactin sutures. Two or three fornix fixation sutures were used to deepen the fornix of the 4 − 0 absorbable braided polyglactin sutures. The conformer was placed in the socket with temporary tarsorrhaphy using 5 − 0 silk sutures for at least 6 weeks postoperatively. The surgical technique is demonstrated in Fig. 1.

The outcome was considered successful if the following criteria were met: (1) patients could wear their eye prosthesis without experiencing displacement [3] and (2) absence of re-operation or additional surgery after socket reconstruction using OMG. Prognostic factors influencing the surgical outcomes were also identified.

The prognostic factors and functional surgical outcomes were analyzed using Pearson’s chi-squared test, and factors that could influence the success of functional outcomes were analyzed using Fisher’s exact test. Univariate logistic regression was used to evaluate the factors influencing the postoperative outcomes. Stepwise backward logistic regression was used to identify factors associated with successful surgical outcomes. Age and sex were retained irrespective of their statistical significance. Statistical significance was set at *p*-value < 0.05.

## Results

This study included 37 patients. The demographic data and clinical characteristics before anophthalmic socket reconstruction using autologous OMG are shown in Table 1. All patients who underwent globe removal received volume replacement with an orbital implant. Among the 19 enucleations, the types of orbital implants used were as follows: 4 acrylic balls, 2 glass balls, 1 hydroxyapatite, with data unavailable for the remaining 12 cases. For the 6 eviscerations, 1 acrylic ball was used, and data was unavailable for the remaining 5 cases. The median duration between eye removal surgery and socket contracture was 21.5 years (range: 22 days–61.6 years). Twenty-eight patients (75.7%) underwent socket reconstruction using OMG alone, eight patients (21.6%) underwent reconstruction using OMG combined with a hard palate graft, and one patient (2.7%) underwent reconstruction using a hard palate graft alone. A lateral tarsal strip was performed as co-surgery in 10 patients (27.0%).

**Table 1:** Demographic data and clinical characteristics

Variables	n (%)
**Sex**	
**Male** **Female**	15 (40.5) 22 (59.5)
**Age (years)**	
**Mean (SD)** **Median (min, max)**	40.22 (17.20) 39.50 (8.37, 68.64)
**Age group (years)**	
**<40** **≥40**	20 (54.1) 17 (45.9)
**Reasons for eye removal**	
**Trauma** **Infection** **Tumor** **Others** **NA**	18 (48.7) 5 (13.5) 4 (10.8) 9 (24.3) 1 (2.7)
**Types of eye removal procedure**	
**Enucleation** **Evisceration** **Phthisis bulbi or microphthalmos** **NA**	19 (51.4) 6 (16.2) 7 (18.9) 5 (13.5)
**Duration from eye removal to socket contracture (years)**	
**Mean (SD)** **Median (min, max)**	21.48 (17.09) 23.17 (0.06, 61.61)
**Grading of socket contracture**	
**Grade 2** **Grade 3**	20 (54.1) 17 (45.9)

**Abbreviations**: SD, standard deviation; CI, confidence interval; NA, not available

All donor sites recovered well without serious complications. Among the 37 patients, 25 could wear their eye prostheses without re-operation or additional surgery. The success rates of socket reconstruction in grade 2 and 3 socket contractures were 80.0% and 52.9%, respectively, and the overall success rate was 67.6%. The complications of socket reconstruction surgeries included presence of graft necrosis in three cases (8.1%) and wound dehiscence in three cases (8.1%). Eleven patients (29.7%) required additional surgeries; the most common procedure was a lateral tarsal strip in eight patients. No patients underwent a revised OMG.

At the final visit, 34 patients (91.9%) could wear their eye prostheses. The mean duration of follow-up was 6.3 years (range: 11.0 months–12.6 years). Among the three patients who could not retain eye prostheses at the last follow-up, one could not retain the prosthesis at all after socket reconstruction. However, two patients could wear the prosthesis for 3.5 and 5 years after socket reconstruction, respectively.

**Fig. 2:**
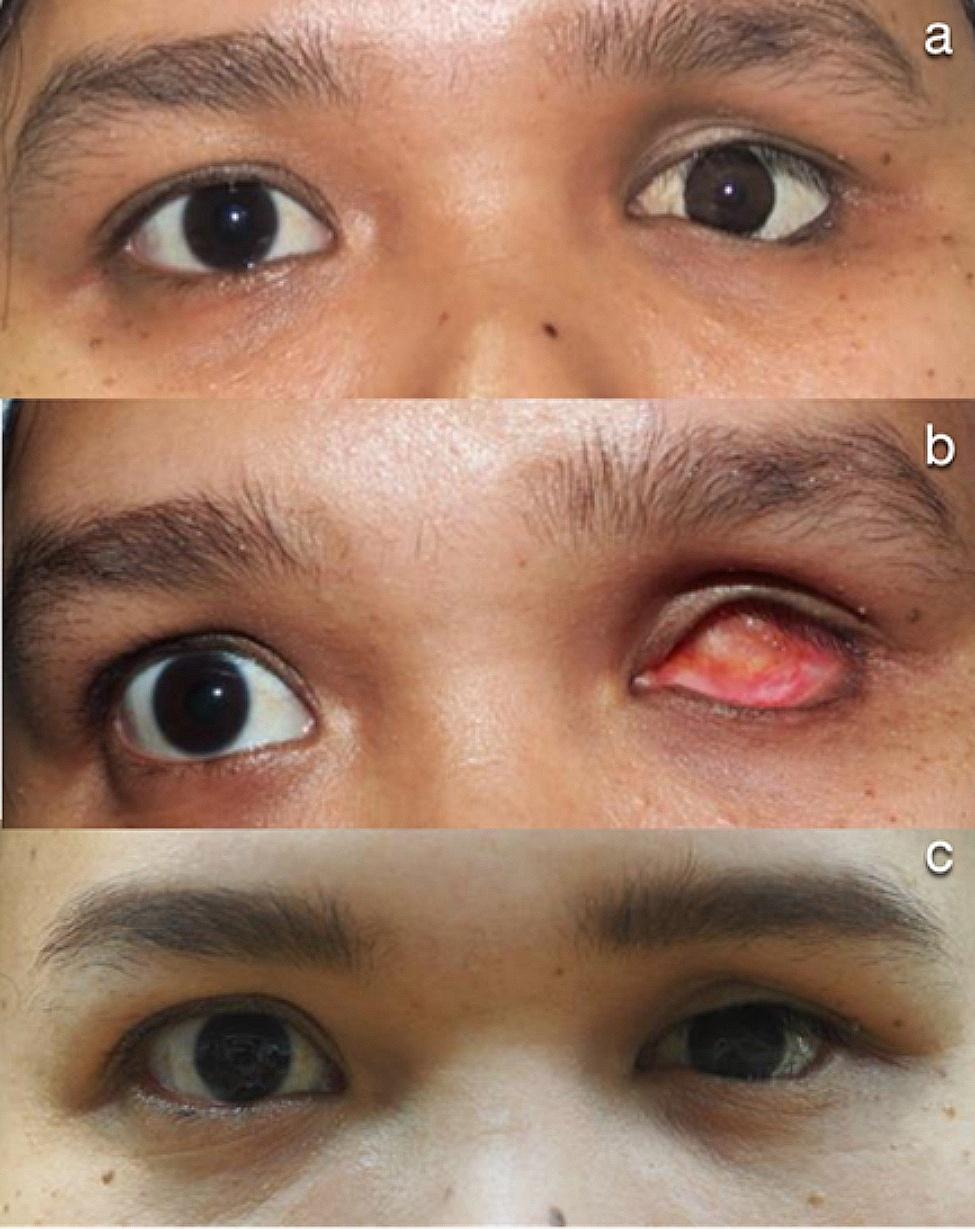
Photographs illustrating patient who underwent socket reconstruction using autologous oral mucosal graft. **a** A 31-year-old woman with a 1-year history of eye prosthesis dislocation and rotation, accompanied by preoperative lower lid sagging in the left eye. **b** Grade 2 socket contracture is depicted. **c** The patient wore eye prosthesis without displacement eight years postoperatively

Univariate analysis demonstrated the variables affecting surgical outcomes (Table 2). When compared to grade 2 socket contracture, grade 3 socket contracture was found to be a prognostic factor associated with less favorable surgical outcomes on multivariate analysis (adjusted odds ratio = 6.04, *p* = 0.040, Table 3). Figure 2 reveals photographs of patient who underwent socket reconstruction using OMG.

**Table 2:** Univariate analysis for successful surgical outcomes

Variables	**Successful outcome**		*p*-value	Successful outcome	*p*-value
	No **n = 12**	Yes **n = 25**		Crude odds ratio (95% CI)	
**Sex**					
**Male**	6 (50.0)	9 (36.0)	0.488	1	
**Female**	6 (50.0)	16 (64.0)		1.78 (0.44, 7.18)	0.419
**Age (years)**					
**Mean (SD)**	42.63 (17.79)	39.06 (17.16)	0.563	0.99 (0.95 1.03)	0.551
**Median (min, max)**	42.31 (8.37, 68.64)	38.90 (8.45, 67.10)	0.561		
**Age group (years)**					
**<40**	5 (41.7)	15 (60.0)	0.482	1	
**≥40**	7 (58.3)	10 (40.0)		0.48 (0.12, 1.93)	0.299
**Reasons for eye removal**					
**Trauma**	5 (41.7)	13 (54.2)	0.428	1	
**Tumor**	1 (8.3)	3 (12.4)		1.15 (0.10, 13.88)	0.910
**Infection**	1 (8.3)	4 (16.7)		1.54 (0.14, 17.33)	0.727
**Others**	5 (41.7)	4 (16.7)		0.31 (0.06, 1.64)	0.167
**Types of eye removal procedure**					
**Enucleation**	4 (36.4)	15 (71.4)	0.153	1	
**Evisceration**	3 (27.3)	3 (14.3)		0.27 (0.04, 1.86)	0.183
**Phthisis bulbi or microphthamos**	4 (36.4)	3 (14.3)		0.20 (0.03, 1.28)	0.090
**Duration from eye removal to socket contracture (years)**					
**Mean (SD)**	25.98 (22.98)	19.32 (13.45)	0.274	0.98 (0.94, 1.02)	0.269
**Median (min, max)**	28.27 (0.9, 61.61)	21.43 (0.06, 46.62)	0.456		
**Contracted socket grading**					
**Grade 2**	4 (33.3)	16 (64.0)	0.157	1	
**Grade 3**	8 (66.7)	9 (36.0)		0.28 (0.07, 1.20)	0.087
**Types of mucous membrane graft**					
**Lip ± buccal mucosa**	8 (66.7)	20 (80.0)	0.407	1	
**Hard palate**	0 (0.0)	1 (4.0)		1 (omitted)	-
**Combined grafts**	4 (33.3)	4 (16.0)		0.40 (0.08, 2.00)	0.265

**Abbreviations**: SD, standard deviation; CI, confidence interval

**Table 3:** Multivariate analysis for successful surgical outcome

Variables	Successful outcome	*p*-value
	Adjusted odds ratio (95% CI)	
**Sex**		
**Male**	1	
**Female**	5.82 (0.75, 45.18)	0.092
**Age (years)**	1.03 (0.97, 1.10)	0.308
**Duration from eye removal to socket contracture (years)**	0.93 (0.86, 1.00)	0.058
**Grading of socket contracture**		
**Grade 3** **Grade 2**	1 6.04 (1.09, 33.52)	0.040

Abbreviations: CI, confidence interval

## Discussion

After evaluating patients with grade 2 and 3 anophthalmic socket contractures who required OMG to replace the conjunctiva and reconstruct the fornices, we found that grade 3 contracture was a predictive factor associated with inferior surgical outcomes. Additionally, for every one-year increase in the duration from eye removal to socket contracture, there was a potential 7% decrease in the success rate of eye prosthesis fitting, with a *p*-value of 0.058.

The primary aim of surgical management of socket contracture is to form a fornix that can maintain the eye prosthesis and simulate a normal fellow eye. The general principle of socket reconstructive techniques is excision of the conjunctival scarred tissues, followed by graft or flap placement to replace lost conjunctiva and enlarge the forniceal space [2]. Several grafts are mentioned in the literature, including oral mucosa, [2, 3, 7] hard palate, [6, 8] skin graft, [9, 10] dermis fat graft, [11] and polytetrafluoroethylene [12, 13]. Previous studies have reported that using a full-thickness mucous membrane graft, which allows the grafted tissue to match the conjunctiva histologically, is successful in almost all degrees of contractions [3, 14, 15]. 

The principal issue of this study was the foreshortening of the conjunctival fornices, apart from orbital volume loss. Various graft materials have been used for socket reconstruction to deepen fornices, and each presents with its own advantages and disadvantages. Amniotic membranes are easy to use but lack the rigidity to maintain the fornices. Additionally, the amniotic membrane has a high rate of recurrent socket contracture. Since it is similar to normal conjunctiva, it is more suitable for mild degrees of socket reconstruction [2, 16]. Full-thickness skin grafts are easy to harvest in large areas to reconstruct severe socket contractures with a high success rate [10]. However, the disadvantage of full-thickness skin grafts is keratin production, which affects chronic socket discharge. Since OMG has biological properties similar to those of the conjunctiva, it is the most popular for managing foreshortened conjunctival fornices. However, the disadvantages of OMG include limited harvesting areas, donor site complications, and lack of rigidity.

The overall success rate of socket reconstruction using OMG in our study was lower than that reported in previous studies [3, 17]. The possible reasons for this could be as follows. First, our study had a longer follow-up period (6.3 years) than previous studies, which had short follow-up periods of 8.7 months to 3.5 years [3, 17]. Second, we used strict criteria to define successful outcomes that could possibly represent the natural course of anophthalmic socket change after reconstruction. Approximately one-third of all patients in our study required additional surgery during the follow-up period. In grade 2 and 3 contractures, the surgical approach for socket reconstruction involves an autologous mucous membrane graft. However, grade 3 contractures show a more extensive area of conjunctival shrinkage than grade 2, and patients with grade 3 socket contractures cannot wear an eye prosthesis. Normally, larger OMG harvesting is required for patients undergoing grade 3 socket reconstruction, and reducing the risk of further socket contracture in these patients may increase the success rate of wearing eye prostheses. Intraoperative topical application of mitomycin C (MMC), [18] topical MMC after socket reconstruction, [19] and subconjunctival 5-fluorouracil [20] are the augmented-treatment modalities used to prevent scar formation. MMC and 5-fluorouracil are antimetabolites that inhibit fibroblast proliferation and may be beneficial in achieving better outcomes.

The most common additional surgery in our study was the lateral tarsal strip, which eliminated lower laxity and increased the chance of eye prosthesis retention. This result may suggest that intensive lower lid evaluation in consideration of the lateral tarsal strip as a co-operation in case of lower lid laxity is important. Therefore, this concept warrants further investigation. Although patients in our study who met the criteria for a successful surgical outcome generally experienced favorable results, some might have had sub-optimal outcomes. Nonetheless, these patients expressed satisfaction with their postoperative condition.

The strength of our study was that we enrolled only patients with grade 2 or 3 socket contractures who required socket reconstruction using OMG. Patients with grade 4 contracture, including those with a history of radiation and multiple previous socket surgeries, were not enrolled. However, our study has a few limitations, including its retrospective nature. Some data might have been lost owing to possible recording system errors and lack of details on objective measurements, such as the height of the fornix, the lower lid laxity, and the degree of socket moisture. In addition, this study evaluated only functional outcomes and did not include cosmetic outcomes. Owing to the insufficient follow-up period, we suggest that further studies evaluate postoperative outcomes in the long term since socket contracture continues even after reconstruction surgery.

## Conclusions

In summary, grade 2 socket reconstruction using autologous OMG has better surgical outcomes than grade 3 socket reconstruction. This procedure can be safely performed without serious donor site complications. Preoperative severe socket contracture is the only factor associated with worse surgical outcomes, and the use of augmented antimetabolites can be beneficial in preventing postoperative scarring and increasing the possibility of wearing an eye prosthesis.

## Data Availability

All unidentifiable data are available from the corresponding author upon reasonable request.

## Abbreviations

OMG: Oral mucosal graft
MMC: mitomycin C
